# Influence of Burnout and Feelings of Guilt on Depression and Health in Anesthesiologists

**DOI:** 10.3390/ijerph17249267

**Published:** 2020-12-11

**Authors:** Alejandra Misiolek-Marín, Ana Soto-Rubio, Hanna Misiolek, Pedro R. Gil-Monte

**Affiliations:** 1 Clínica Art Psychology and Psychotherapy, Calle Diputación 153, 08011 Barcelona, Spain; aleksandramisiolek@gmail.com; 2 Department of Personality, Assessment and Psychological Treatments, Faculty of Psychology, University of Valencia, Av. Blasco Ibáñez, 21, 46010 Valencia, Spain; 3 Department of Anesthesiology and Intensive Therapy, Medical University of Silesia in Katowice, Poniatowskiego 15, 40-055 Katowice, Poland; hanna.misiolek@gmail.com; 4 Department of Social Psychology, Faculty of Psychology, Unidad de Investigación Psicosocial de la Conducta Organizacional (UNIPSICO), University of Valencia, Av. Blasco Ibáñez, 21, 46010 Valencia, Spain; pedro.gil-monte@uv.es

**Keywords:** burnout, depression, psychosomatic health, anesthesiologists

## Abstract

Background and objective: The WHO has included burnout as an occupational phenomenon in the ICD-11. According to the WHO, burnout is a syndrome conceptualized as resulting from chronic workplace stress that has not been successfully managed. The study aimed to evaluate the influence of feelings of guilt and burnout on health in Polish anesthesiologists. Alcohol and tobacco intake, psychosomatic disorders, and depression were assessed. Methods: The study had a non–randomized cross-sectional character. The sample consisted of 372 Polish anesthesiologists. Burnout was measured by the Spanish burnout inventory. Results: Post hoc analysis for burnout consequences: depression (F_(5,366)_ = 17.51, *p* < 0.001, η_p_^2^ = 0.193), psychosomatic disorders (F_(5,366)_ = 13.11, *p* < 0.001, η_p_^2^ = 0.152), and tobacco intake (F_(5,366)_ = 6.23, *p* < 0.001, η_p_^2^ = 0.078), showed significant differences between burnout with and without the highest levels of feelings of guilt. All the instruments applied were reliable. Conclusions: Depression, psychosomatic disorders, and alcohol and tobacco intake are suspected to be consequences of the highest guilt levels related to burnout, i.e., Profile 2 according to the burnout model of Gil-Monte. Participation in prevention programs is recommended for these cases.

## 1. Introduction

Chronic stress may lead to many negative consequences, such a burnout. Studies show that psychological distress is significantly associated with burnout [1]. Doctors are exposed to high-stress levels in their profession and are particularly susceptible to experiencing burnout [2,3,4]. The levels of burnout are high in medical personnel than in other professionals and tend to increase over the years. Satisfaction with work–life balance in US physicians worsened from 2011 to 2014, and more than half of US physicians are now experiencing professional burnout [5]. However, there is no consensus regarding the general prevalence of burnout in health professionals [6].

The World Health Organization (WHO) has included burnout as an occupational phenomenon in the 11th Revision of the International Classification of Diseases (ICD-11) [7]. It is not classified as a medical condition. According to the WHO, burnout is a syndrome conceptualized as resulting from chronic workplace stress that has not been successfully managed. It refers specifically to phenomena in the occupational context and should not be applied to describe experiences in other areas of life [7].

## 2. Background

### 2.1. Burnout in Anesthesiologists

Burnout levels in anesthesiologists are reported to be either very high in comparison to other medical specialties (40% of anesthesiologists are at risk of burnout according to Nyssen and Hansez [8]) or burnout prevalence greatly varies across studies: 10–41% high risk, up to 59% at least moderate risk, according to a recent systematic review (Sanfilippo et al., 2017). Mikalauskas et al. [9] found that overall, 42% of participants in a study of burnout in anesthesiologists were at risk of professional burnout.

Anesthesiologists were chosen to be studied because this specialty is considered one of the most stressful compared to other medical specialties in Poland [10,11,12] and worldwide [13]. Anesthesiologists carry high responsibilities during surgery and may frequently face stressful scenarios such as managing unanticipated difficult airways, cardiac arrest, and other life-threatening emergencies [13]. In Poland, Anesthesiology includes intensive care and the severe state of their patients and frequently lack of follow up; intubation; assessing brain death; informing the families of the patients; necessity to take fast decisions; work under pressure; high workload and shortage of personnel; low salary and a necessity to work in various hospitals simultaneously; long working hours; exhaustion; lack of time for friends and family; lack of proper teamwork with other physicians, especially surgeons; and a lack of social prestige as compared to other specialties, were among the stress factors indicated by the anesthesiologists in the survey [12].

Van der Walt [14] mentions the negative consequences of high burnout in anesthesiologists. He emphasizes that the risk of suicide and drug dependence is twice much higher in this group as in the rest of the population.

### 2.2. The Burnout Model

Gil-Monte [15] described burnout as a psychological response to chronic work-related stress of interpersonal and emotional nature. This response is characterized by cognitive deterioration (i.e., loss of enthusiasm towards the job) and emotional deterioration (i.e., emotional and physical exhaustion), which lead to negative attitudes and behaviors towards patients as a coping strategy (e.g., indifference, cynicism, indolence, and withdrawal). In some cases, feelings of guilt appear due to negative attitudes developed toward the job, and guilt appears to be involved in the burnout syndrome [16,17,18]. One of the frequent causes of guilt in professionals is negative thoughts about others and the negative and cynical way they have treated them. Some professionals underestimate the influence of situations on behavior and interpret their experiences as reflecting some personality malfunction, leading them to blame themselves for not performing their job adequately. As a result, they develop a sense of failure and loss of self-esteem [17] (pp. 5, 10–12). These professionals could feel that they are becoming cold and dehumanized. This experience leads them to reaffirm their commitment toward other people and the responsibility of taking care of them [19], resulting in higher levels of burnout. Some burnout models that describe the process have identified feelings of guilt as one of the most destructive factors in staff burnout [18].

Gil-Monte [15,20] integrated the feelings of guilt into a theoretical model to explain different burnout profiles to reach a complete diagnosis, identify individuals affected by the syndrome, and recognize the syndrome’s influence on health-related disorders. In this model, guilt is considered a dimension of burnout. It explains the distinction between Profile 1 and Profile 2, characterized by higher feelings of guilt that may appear and lead to serious health consequences (e.g., depression, excessive smoking, alcohol abuse, etc.) [20,21].

The professionals with Profile 1 will not often feel guilty because of treating patients without consideration or aggressively or not adjusting to their role expectations. These professionals can persist in the organization for years without developing relevant personal problems concerning work-related stress. However, with their attitude and behavior of indifference, apathy, irresponsibility, cynicism, indolence, and so on, they deteriorate the quality of the organization’s service and give rise to complaints from patients about the way they are treated.

On the contrary, the professionals who develop Profile 2 experience higher remorse because of not fulfilling their role adequately, because of feeling exhausted, and treating the patients impersonally and with indolence. As stressful working conditions do not change, there is a loop over time that produces a dysfunctional and disruptive experience and symptoms of depression [20]. These profiles can be estimated by the Spanish burnout inventory (SBI). This instrument is based on a theoretical model created prior to a psychometric one, and it overcomes the theoretical and psychometric limitations of other instruments [22].

Although the model of the Maslach burnout inventory (MBI) [23] has been the dominant paradigm in research on the processes underlying burnout, some alternative models have hypothesized different types of burnout that coincide more closely with the clinical experience. Currently, it has been accepted that burnout syndrome can be differentiated into profiles or types of burnout [24,25,26,27,28,29] and that high scores in any of those types or profiles—i.e., high burnout—do not necessarily assume that professionals who score high have disorders of health. For example, Tops et al. [28] distinguished burnout subjects with high basal prolactin levels—prolactin profile—versus low basal prolactin levels. Burnout subjects in prolactin profile had high scores on depression, but burnout subjects showing low prolactin levels were characterized by low attachment/high distance, and more severe state negative affect and anxiety. Van Dam [29] distinguished between mild symptom group burnout, a subclinical group of burnout with fatigue as the most prominent symptom, and a severe burnout group, with anxiety and depression levels psychiatrically relevant. Oosterholt et al. [26] distinguished between clinical and non-clinical burnout groups considering the cognitive problems reported.

In some models on the process of burnout, feelings of guilt have been identified as one of the most destructive factors in staff burnout [18,30]. The SBI model integrates feelings of guilt into a theoretical model to explain different burnout profiles (Profile 1 vs. Profile 2) to reach a complete diagnosis, identify individuals affected by the syndrome, and recognize the syndrome’s influence on health-related disorders. Taking into consideration the model of the SBI [15,20,22], Profile 1 would be similar to the low basal prolactin [28] cynicism cluster [24], disengaged profile [25], non-clinical burnout [26], or mild symptom group [29]. On the other hand, Profile 2 would be in line with the prolactin profile [28], burnout cluster [24], clinical burnout [26], or severe burnout group [29].

### 2.3. Burnout and Depression

Burnout may be a phase in the development of work-related depression [31]. It can co-occur with anxiety and, in the later stages, be accompanied by depressive symptoms [32].

The association of burnout with depressive symptoms has been confirmed in longitudinal studies on Finnish dentists [33,34]. However, burnout represents a separate diagnostic entity rather than a form of depression [35]. In a recent meta-analysis [36] regarding an overlap between burnout and depression, the results showed that burnout and depression are associated. However, the effect size is not strong enough to suggest that they are the same construct, suggesting that burnout and depression are two different constructs rather than one. In our study, we assumed depression is a separate construct and a consequence of burnout on health.

According to some authors, it is challenging to distinguish correctly between them [37,38]. However, the distinction between burnout and depression is partly supported by empirical research. Most factoring studies concluded that burnout could be psychometrically distinguished from depression [37]. Glass and McKnight [39], in a review of 18 burnout and depression studies, showed that both syndromes have many common features (variation 15–20%). Although burnout and depression are positively correlated, they seem to be two distinct constructs [40] and distinct phenomena.

Poncet et al. [41], in their study of burnout on nurses, confirmed that 12% of the studied group suffered from high burnout and depression simultaneously, and only 4% suffered from depression without suffering from high burnout. Embriaco et al. [42] studied intensivists. They confirmed that 46.65% of the studied group showed high burnout levels, 24% suffered from depression, but only 80.6% of the intensivists exhibiting symptoms of depression presented a high level of burnout.

According to the theoretical model of Gil-Monte [15], one reason for these results could be the existence of profiles of burnout. While Profile 1 does not lead to developing depression, Profile 2 of burnout leads to negative health consequences, such as depression [20].

### 2.4. Burnout and Health Disorders

Psychosomatic health, defined as physical problems resulting from emotions [43], is considered to be affected in burned-out physicians and anesthesiologists [44,45]. The comorbidity of burnout and health consequences such as depression and other medical staff’s psychological problems has been confirmed in various studies. Trufelli et al. [46] observed that 13–44% of medical personnel suffered from other psychological problems apart from burnout. Studies on hospital employees’ health in Swedish nurses reveal that 8% of labor health problems are related to burnout [47].

Burnout may also lead to negative consequences for society, as it is the reason for inadequate patient’s treatment and medical errors. In a study on medical personnel in Hungary [48], psychosomatic health problems resulting from burnout were analyzed. It has been proved that emotional exhaustion is a predictor of psychosomatic disorders. Reviewing the literature, Lapa et al. [49] found that sleep disorders, increased cardiovascular risk, diabetes, obesity, acceleration of the rate of biological aging, alcoholism, and drug addiction, and suicide ideation were between the consequences of burnout in anesthesiologists and physicians. Additional consequences were identified by Campos and de Barros [50]: fatigue, headaches, increase in blood pressure and heart rate, sleep disorders and eating disorders, and emotional problems. Mikalauskas et al. [9] found significant associations between burnout and alcohol dependence, duration of sleep, cardiovascular disorders, and digestive disorders in a sample of anesthesiologists and intensive care physicians.

Maslach [51] pointed out that the issue of the relation between burnout and psychological health is complex and emphasizes that in many studies, burnout was linked with neurotic traits and with work-related neurasthenia [52,53]. At the same time, it should be considered that psychiatric disorders such as anxiety [54] and depression [20,33] should be considered as consequences of burnout [29]. On the other hand, some researchers argue that psychologically healthy persons cope better with chronic stressors and less frequently suffer from burnout [55,56].

Although it has been shown that burnout syndrome has a negative influence on health, not much attention has been paid to this problem, as compared to the number of studies on the negative consequences of stress. For example, there are many publications on alcoholism and smoking because of stress but very few on smoking due to burnout. According to Avery et al. [57], 10–15% of physicians are addicted to psychoactive substances. The study confirms that the intake of such substances increases due to a stressful situation, especially work-related stress. Therefore, stressful work may lead to adverse health consequences such as alcohol abuse rather than alcohol abuse may be a predictor of burnout. In anesthesiologists, burnout syndrome may lead to problems like alcoholism and substance abuse [58].

This study aimed to analyze, according to the theoretical model of Gil-Monte [15,20], the influence of burnout and feelings of Guilt (Profile 2) on the levels of depression, psychosomatic disorders, and alcohol and tobacco intake in a sample of Polish anesthesiologists. The hypothesis of the study were as follows:

**Hypothesis** **1.** *Participants of Profile 2 will significantly show the highest levels of depression, and these levels will be significantly higher than participants of Profile 1.*

**Hypothesis** **2.** *Participants of Profile 2 will significantly show the highest levels of psychosomatic disorders, and these levels will be significantly higher than participants of Profile 1.*

**Hypothesis** **3.** *Participants of Profile 2 will significantly show the highest levels of alcohol intake, and these levels will be significantly higher than participants of Profile 1.*

**Hypothesis** **4.** *Participants of Profile 2 will significantly show the highest tobacco intake levels, and these levels will be significantly higher than participants of Profile 1.*

## 3. Methods

The study was approved to be conducted by the Bioethical Commission of the University of Silesia, and it did not require the assessment of the Commission due to its non-experimental character. All procedures performed in studies involving human participants were following the ethical standards of the institutional or national research committee and with the 1964 Helsinki declaration and its later amendments or comparable ethical standards.

### 3.1. Measures

The levels of burnout were measured by the Spanish burnout inventory (SBI) [22] in its Polish adaptation [59,60]. The adaptation of the SBI scale showed adequate values of factorial analysis. The four-factor model obtained a good fit for the sample: χ^2^_(164)_ = 324.04 (*p* < 0.001), χ^2^/df = 1.98, RMSEA = 0.051 (90% confidence intervals: 0.043–0.059), GFI = 0.920, NNFI = 0.940, CFI = 0.949. All of the factor loadings were statistically significant for *p* < 0.001.

The instrument is a 20–item scale distributed into four subscales: (1) enthusiasm towards the job, defined as the individual’s desire to achieve goals at work as a source of personal pleasure (5 items; e.g., I find my work is a stimulating challenge, Cronbach’s alpha = 0.86); (2) psychological exhaustion, defined as the appearance of emotional and physical exhaustion due to the necessity to deal with people with problems (4 items; e.g., I feel emotionally exhausted, Cronbach’s alpha = 0.85); (3) indolence, defined as appearance of negative attitudes of indifference and cynicism towards the patients (6 items; e.g., I feel like being sarcastic with some patients, Cronbach’s alpha = 0.80); and (4) guilt, defined as the appearance of the feelings of guilt about the negative attitudes towards patients and workplace (5 items; e.g., I regret some of my behaviors at work, Cronbach’s alpha = 0.86).

According to the SBI Manual, global burnout comprises values on the mean of the 15 items from the subscales of enthusiasm towards the job (reversed; 5 items), psychological exhaustion (4 items), and indolence (6 items; Cronbach’s alpha = 0.84). Low levels of enthusiasm toward the job with high levels of psychological exhaustion and indolence are found in Profile 1. Higher feelings of guilt account for the difference between Profile 1—i.e., high global burnout and low feelings of guilt—and Profile 2—i.e., high global burnout and higher feelings of guilt (see Table 2, Note 1).

Psychosomatic disorders were measured by a 9-item scale of the UNIPSICO questionnaire [58,59]. Items include different work-related psychosomatic disorders (e.g., headaches, musculoskeletal pain, sleep quality, anxiety, and sickness; e.g., headaches, musculoskeletal pain, sleep quality, anxiety, illness; e.g., Do you have a headache?; Cronbach’s alpha = 0.83). Alcohol and tobacco use was evaluated using one item that alluded to the frequency with which these products had been used during recent weeks as a consequence of troubles related at work: “As a consequence of troubles related at work, Has your daily alcohol use increased?”; “As a consequence of troubles related at work, Has your daily tobacco use increased?”. Participants answered the items on all the subscales on a 5-point frequency scale ranging from “Never” (0) to “Very frequently: Every day” (4). All these scales were translated into Polish using the back-translation method.

Depression was estimated by the Polish adapted version of Beck’s depression scale [60]. This instrument is a 21-question multiple-choice self-report inventory and consists of 21 items. The items were answered on a four-point scale, ranging in frequency from 1 (A little of the time) to 4 (Most of the time; Cronbach’s alpha = 0.89). The level of depression is a sum of all points.

### 3.2. Procedure

The study had a cross-sectional design. The study was conducted in 2013, and the data were collected in a non-random way in the Northern, Central, and Southern Polish public hospitals. Heads of Anesthesiology Departments of non-randomly selected hospitals in Southern, Central, and Northern Poland were contacted and asked to distribute questionnaires among the anesthesiologists. Six hundred questionnaires were distributed among anesthesiologists (approximately 15% of all Polish anesthesiologists). Participation was voluntary and anonymous. Completed surveys were put each into a sealed envelope with a return stamp by the participant, guaranteeing confidentiality, and send back to the researchers. The participants were informed that by filling in the questionnaires they agreed to participate in the study.

### 3.3. Statistical Analysis

The theoretical model of the SBI distinguishes between two types of burnout (Profile 1 vs. Profile 2). The appearance of critical levels of guilt—i.e., values equal to or higher than the 90th percentile in the subscale of guilt—account for the difference between Profile 1—i.e., professionals who do not develop high feelings of guilt—and Profile 2. In Profile 1, participants obtained values equal to or higher than the 90th percentile—according to SBI Manual values, Gil-Monte [22]—on the mean of the 15 items from the subscales of enthusiasm towards the job (reversed; 5 items), psychological exhaustion (4 items), and indolence (6 items), and values lower than the 90th percentile on the guilt subscale. In Profile 2, participants met the criteria of obtaining values equal to or higher than the 90th percentile on the mean of the 15 items, together with values equal to or higher than the 90th percentile on the guilt subscale.

The program IBM SPSS Statistics v.21 was used for the statistical analysis. Cronbach’s alphas were used to assess the reliability of the scales. To compare variables, the analysis of variance (ANOVA) and post hoc Bonferroni test were used. The outliers were analyzed, and the ones that were aberrant and not representative of any observations in the population were eliminated. One case was eliminated because it showed the z value very high as for the depression variable (z = 6.68).

## 4. Results

Three hundred and seventy two professionally active anesthesiologists of Polish hospitals participated in the study (62% returned questionnaires from the 600 originally sent). The studied groups consisted of 57.8% (*n* = 215) women and 42.2% (*n* = 157) men. Age, M = 42.05 (*SD* = 9.99, min. = 26, max. = 65); seniority in the position, M = 11.23 (*SD* = 9.21, min. = 1, max. = 39); seniority in the profession, M = 16.16 (*SD* = 10.12, min. = 1, max. = 40); and seniority as a head of department, M = 8.92 (*SD* = 7.47, min. = 0, max. = 39).

Cronbach’s alpha results to assess the reliability of the scales are presented in Table 1. According to Cronbach’s alpha, the reliability of all scales was higher than the minimum 0.70, confirming good reliability of the scales. The measures used to evaluate the variables fitted the normal distribution to a great extent because the skewness values ranged between −2 and 2. Only the skewness value for tobacco slightly exceeded the value of +2, which means that this deviation was not important. The kurtosis value for depression slightly exceeded the value of +2. The value for tobacco slightly exceeded the value of +4, which means that this deviation is not relevant, considering the sample size [61,62].

Psychosomatic disorders, excessive intake of tobacco and alcohol, and depression considered as consequences of burnout were analyzed in relation to the levels and profiles of global burnout and SBI subscales. Table 2 shows the ANOVA taking into consideration the burnout levels. Significant differences between the groups were observed considering the different burnout levels for all variables considered as consequences. The highest burnout level group had shown the highest level of consequence subscale. Profile 2 are values equal to or higher than the 90th percentile on global burnout together values equal to or higher than the 90th percentile on the guilt subscale.

**Figure 1:**
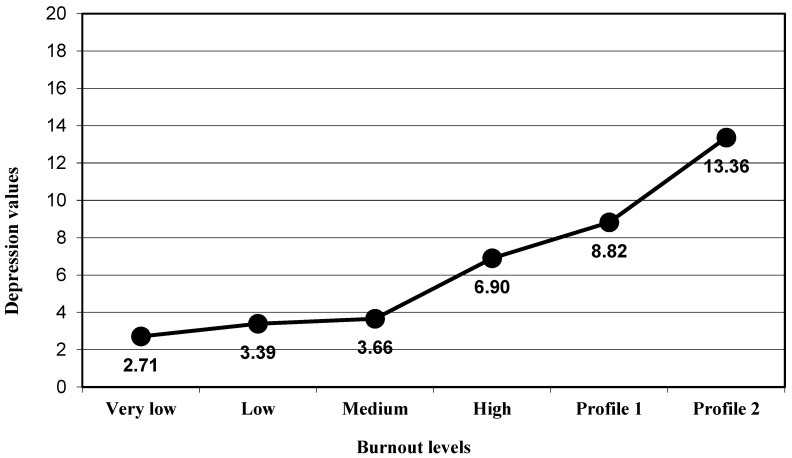
Mean in the depression variable for the groups of burnout levels.

**Figure 2:**
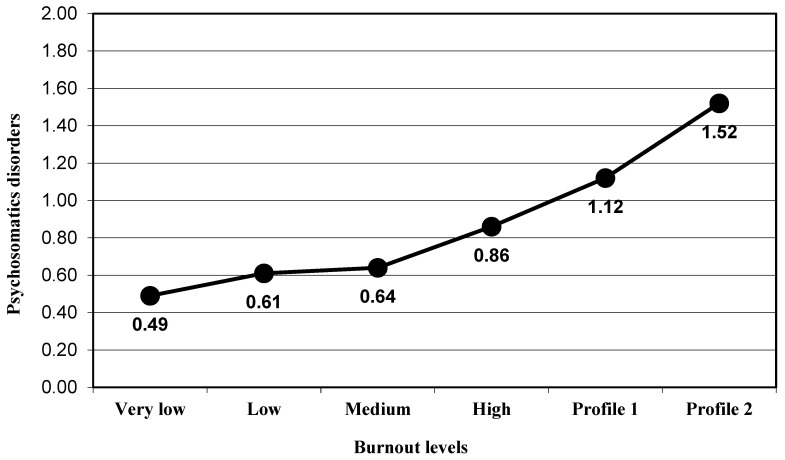
Mean in the psychosomatic disorders variable for the groups of burnout levels.

**Figure 3:**
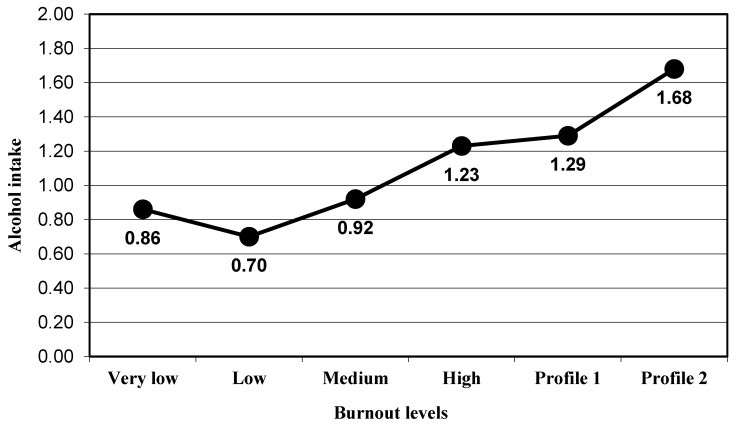
Mean in the alcohol intake variable for the groups of burnout levels.

**Figure 4:**
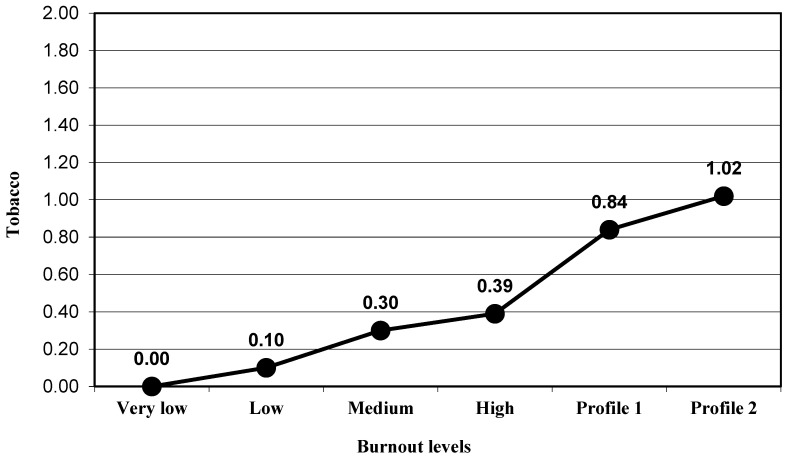
Mean in the tobacco use variable for the groups of burnout levels.

The participants presented a significant progressive increased in the depression levels (group very low, M = 2.71 to group Profile 2, M = 13.36) when the global burnout levels increased (F_(5,366)_ = 17.51, *p* < 0.001, η_p_^2^ = 0.193; Figure 1). With regard to psychosomatic disorders, a significant change was also obtained (F_(5,366)_ = 13.11, *p* < 0.001, η_p_^2^ = 0.152; Figure 2). ANOVA results were significant and in the expected direction for alcohol intake (F_(5,366)_ = 4.18, *p* < 0.001, η_p_^2^ = 0.054; Figure 3) and for tobacco use (F_(5,366)_ = 6.23, *p* < 0.001, η_p_^2^ = 0.078; Figure 4; Table 2).

Taking into consideration the post hoc analysis for the variable depression, the results showed that there was a significant difference in depression levels between the participants included in the high levels of burnout group (M = 6.90) and participants in Profile 2 (M = 13.36; *p* < 0.001). The difference was not significant for the difference between the high levels of the burnout group and participants in Profile 1 (M = 8.82; *p* = 0.635), and the difference between participants in Profile 1 and participants in Profile 2 was significant (*p* = 0.025; Figure 1). These results confirmed Hypothesis 1.

Regarding the post hoc analysis for the variable psychosomatic disorders, the results showed that there was a significant difference in psychosomatic disorders levels between the participants included in the high levels of burnout group (M = 0.86) and participants in Profile 2 (M = 1.52; *p* < 0.001). The difference was not significant for the difference between the high levels of burnout group and participants in Profile 1 (M = 1.12; *p* = 0.113). The difference between participants in Profile 1 and participants in Profile 2 was not significant (*p* = 0.149; Figure 2).

For the variable alcohol intake associated with occupational problems, the results showed significant differences between the participants included in the medium levels of the burnout group (M = 0.92) and participants in Profile 2 (M = 1.68; *p* = 0.023), and between the participants included in the low levels of the burnout group (M = 0.70) and participants in Profile 2 (*p* = 0.005). The difference was not significant between the medium levels of the burnout group and participants in Profile 1 (M = 1.29; *p* = 0.630) nor between the participants included in the low levels of the burnout group and Profile 1 (*p* = 0.125). Besides, no significant differences were obtained for the differences between other groups (Figure 3).

Finally, for the variable tobacco use associated to occupational problems, the results showed significant differences between the participants included in the high levels of the burnout group (M = 0.39) and participants in Profile 1 (M = 0.84; *p* = 0.031), and participants in Profile 2 (M = 1.02; *p* = 0.022; Figure 4).

According to these results, Hypothesis 2, Hypothesis 3, and Hypothesis 4 were partially confirmed as the participants presented a significant progressive increase in the psychosomatic disorders and alcohol and tobacco use levels respectively, when the global burnout levels increased. However, no significant differences were obtained for the differences between participants in Profile 1 vs. Profile 2 for those variables.

## 5. Discussion

This study aimed to analyze the influence of burnout levels and feelings of guilt, as a symptom of burnout, on the levels of depression, psychosomatic disorders, and alcohol and tobacco intake, considering the model of Gil-Monte [15,20]. According to our results, adverse health consequences of burnout—i.e., depression, psychosomatic disorders, and alcohol and tobacco intake—significantly increase when individuals feel critical guilt levels. Participants in Profile 2 group—i.e., values equal to or higher than the 90th percentile on global burnout together with values equal to or higher than the 90th percentile on the guilt subscale—showed the highest levels on negative consequences of burnout.

Additionally, participants in Profile 2 scored significantly higher than participants in Profile 1, i.e., values equal to or higher than the 90th percentile on global burnout together with values lower than the 90th percentile on the guilt subscale, for the variable depression. It seems that the feelings of guilt are, therefore, the intermediary element that links burnout with high symptoms of depression [20]. As Friberg [63] states, feelings of guilt would put pressure on an individual that can be reduced through their work by helping others, as guilt makes it possible to alleviate the distress produced by a lack of balance in emotional states resulting from social exchanges. However, excessive or inappropriate levels of guilt can produce a dysfunctional and disruptive experience, and psychological and somatization symptoms in some cases [64]. Ghatavi et al. [65] found that individuals with depression problems had a history of guilt feelings. In their conclusions, they suggest the possibility that guilt represents a variable that predisposes individuals to illness.

Regarding psychosomatic disorders, participants in Profile 2 scored significantly higher than participants in the high levels of the burnout group. However, the difference was not significant between participants in the high levels of the burnout group and participants in Profile 1. Although the difference between participants in Profile 2 and participants in Profile 1 was not significant, this result could be explained by the low number of participants in both groups (Profile 1, *n* = 45; and Profile 2, *n* = 22). For the variable alcohol intake associated with occupational problems, the results were more diffuse but in the same direction that the results obtained for the previous variables. Our study assumed that excessive alcohol and tobacco intakes are consequences of critical burnout level and the accompanying feelings of guilt.

Other authors have also studied negative health consequences resulting from high burnout levels and not only burnout itself. Many studies treat the intake of alcohol and other drugs as ways of coping with stress and burnout [9,66]. According to a survey evaluating burnout, health status, depression, reported alcohol and substance use, and social support of anesthesiologists [67], burnout affects health problems, mental health issues, and substance use negatively. It has been concluded that many anesthesiologists exhibit some high-risk burnout characteristics, and these are associated with lower mental health scores. In Poland, a survey conducted on 80 physicians shows that 63% use alcohol as a coping strategy, and almost 50% of them use other drugs [68]. In a study on oncologists and burnout, depression, and anxiety in Brazil [69], 60% had burnout, 12.3% had depression (HADS-D  ≥  11), and 19.4% had anxiety (HADS-A  ≥  11). According to the authors, previous studies conducted with physicians from different countries showed rates of depressive symptoms varying from 8.8% to 28.1%, and 27% of oncologists have psychiatric comorbidities. In a study on anesthesiologists conducted in China, work-related health problems, including insomnia, fatigue, gastric ulcer, and hypertension, were common and mental problems like drug addiction, depression, and even a tendency to commit suicide are mentioned frequently [70].

However, our study adds the analysis of the possible connection factor between burnout and the negative health consequences, which is the feeling of guilt. Overall, the results offer support to conclude that guilt is a relevant variable in explaining the development of burnout and its influence on health, indicating that guilt feelings contribute to explaining different forms of the evolution of burnout linked to the development of guilt [20,21]. Tops et al. [28] distinguished between burnout individuals with high basal prolactin levels, prolactin profile, vs. low basal prolactin levels. According to the study results, the low prolactin burnout participants scored higher on state negative affect measures, suggesting an essential role of decreased dopaminergic functioning in burnout. This role is similar to the relationship between higher feelings of guilt, depression, and dopamine deficiency [71]. Contemporary burnout theories have stated that physiological changes in the dopaminergic/motivational system due to overriding fatigue for prolonged periods may be fundamental in disorders like burnout [72].

Although our study is a cross-sectional study and does not allow one to conclude on the cause–effect relationship, it may suggest that there is one and further longitudinal studies should be conducted. According to the applied questionnaire, the feeling of guilt is considered part of the burnout syndrome and is also one of the symptoms of depression. Nevertheless, there are cases of anesthesiologists who suffer from critical burnout and do not suffer from depression and who suffer from critical burnout and other health consequences like tobacco or alcohol use but not from depression. Therefore, our results are in line with the results of Koutsimani et al. [36], and they contribute to concluding that critical burnout and depression are not the same construct.

Profile 2, according to SBI, could be understood as a bridge between burnout and depression. Professionals in this level of burnout feel remorse and reaffirm their commitment toward patients and relatives as a restorative behavior to alleviate emotional distress. On the contrary, for the professionals on Profile 1, indolence can be understood as a coping strategy that arises to handle the perception of low enthusiasm toward the job and high psychological exhaustion levels. This coping strategy is sufficient for them to manage the levels of strain. Professionals who develop aggression blame others for difficulties and problems, and resentment toward them is less restrained [17] (p. 12), in contrast to professionals who develop feelings of guilt and depression because they blamed themselves. Individuals fitting Profile 2 will develop high commitment in their jobs, helping others as a way to reduce feelings of guilt [19] and alleviate the emotional distress resulting from taking responsibility for causing others’ suffering. However, as stressful working conditions do not change, there is a loop over time that produces a dysfunctional and disruptive experience, and later disorders of health, i.e., depression or a depressive disorder.

Additionally, the study results give support to recommend the 90th percentile and Profile 2 as a cut-off to differentiate between individuals with severe levels of burnout as a syndrome evaluated by the Spanish burnout inventory [22]. It has been conducted on a large homogenous group of anesthesiologists around Poland, which indicates its high generalizability.

## 6. Limitations and Future Lines of Research

Some limitations of the study should also be acknowledged. The main one is its non-randomized and non-experimental character. This fact implies that there is a possibility that the anesthesiologists who accepted to participate in the study are those who present less burnout in general, being, therefore, more motivated and willing to participate in this type of study and complete and send the questionnaires. Perhaps this has left out of the study those professionals with the highest levels of burnout and who, therefore, presented more motivational difficulties to participate in this type of study. Therefore, it is possible that the study participants were precisely those anesthesiologists who were most willing to participate and, therefore, possibly had lower burnout levels. That is, the sample would be biased toward anesthesiologists with better health and lower overall burnout levels than the total sample of anesthesiologists. In this sense, the representativeness of the sample is limited. Future research lines could carry out a type of sampling that facilitates the participation of both professionals with low levels of burnout and those with high levels so that the latter are not left out of the study. It would favor the representativeness of the sample. It would provide data, precisely, from the professionals who most need this type of study and in whom the most significant negative consequences of burnout would be observed.

At the same time, a limitation of the present study is that it was carried out in 2013, so it would seem that the data have been outdated. However, while it is true that the profile of burnout in anesthesiologists in Poland may have varied over the years, we believe that the way in which the variables relate to each other remains valid and relevant in studying how guilt is related to burnout and the development of depression and health problems. Although it is true that in 2020 the situation at the health level has changed substantially because of the pandemic caused by COVID-19, we considered that precisely knowing how guilt is related to burnout, depression, and health of health professionals can be of crucial importance in order to care for these professionals at the time they need it most, because we believe that the pandemic will have greatly increased the stressors they face in their daily work [73,74,75].

On the other hand, the study is cross-sectional, making it difficult to confirm the cause–effect relations. Future research lines could carry out studies similar to the one presented here, but of a longitudinal nature, to be able to observe the evolution of the data over time and establish more precise relationships between the variables, especially relationships that enhance the predictive character of the study.

Finally, future research should investigate the processes through which guilt generates positive effects and when it does not. Some studies suggest differences in the relationship of chronic versus predispositional guilt to indices of depression and mental health [76]. It would be interesting to analyze which individual and situational factors cause guilt in the process of burnout and burnout Profile 2.

## 7. Conclusions

In conclusion, the obtained results allowed us to suspect that feelings of guilt (as part of Profile 2 of the burnout) might significantly increase the negative consequences of burnout such as depression, psychosomatic disorders, and alcohol intake. These data are of great importance since they indicate that anesthesiologists who present high levels of burnout not only suffer the direct consequences of burnout, such as demotivation, exhaustion, etc., but also are much more vulnerable to severe psychopathologies such as depression and health problems either by psychosomatic symptoms or indirectly by high alcohol consumption. It is fundamental to have these data present in order to prevent and treat burnout in health professionals, in this case, anesthesiologists, to prevent them from suffering these negative consequences in their physical and psychological health. At the same time, the good functioning of this health sector will be favored, since burnout and its negative consequences not only affect the professionals who suffer from it but also their work performance, which also has repercussions on the quality of the care they provide to patients and their families. These professionals’ well-being is fundamental both for them and for the society that benefits from their good work as professionals. The obtained results suggest that there are anesthesiologists working with patients and suffering from high burnout and its consequences. Therefore, more effort should be made on the part of the organization in order to reduce the causes of burnout, and prevention programs should be implemented.

Regarding both practical and research-oriented contributions, the results of the present study suggest incorporating the evaluation of guilt as a symptom of burnout in order to reach a complete diagnosis, discriminate among subjects affected by the syndrome, and recognize the syndrome’s influence in health-related disorders. Moreover, the relationship between guilt and burnout in the development of pathologies such as depression is especially useful in order to implement prevention plans for the development of psychopathologies in these professionals, since guilt is an element that can be worked on from disciplines such as psychology, which opens the door to intervention that involves not only elements of the environment and working conditions, but also elements that could be worked on with the appropriate psychological support such as guilt and how to cope with it. Therefore, these data are essential in designing and implementing prevention and treatment programs for burnout and its negative consequences on these health professionals. Thus, this study may be an important point of reference for clinicians, facilitating both diagnosis and treatment of different burnout types.

To conclude, it should be noted that for the reasons previously stated, studies such as this one are relevant and necessary, even more so in the current context marked by the health emergency caused by COVID-19 [73,74,75]. The current pandemic accentuates the critical situations with which all health professionals, including anesthesiologists, have to work, and any effort to care for them is needed now more than ever. Elucidating the key elements to prevent the development of burnout and associated pathologies, such as guilt, could contribute to this end.

## Figures and Tables

**Table 1:** Descriptive statistics for scales in the study (*n* = 372).

	Alpha	Mean	*SD*	Min.	Max.	Skewness	Kurtosis
Global Burnout	0.84	1.39	0.52	0.20	3.13	0.29	0.08
Guilt	0.86	0.93	0.57	0.00	4.00	0.76	2.03
Psychosomatic disorders	0.83	0.83	0.62	0.00	2.78	0.90	0.32
Depression	0.89	6.04	6.09	0.00	30.00	1.63	2.94
Alcohol intake	---	1.10	1.04	0.00	4.00	0.52	−0.66
Tobacco	---	0.41	0.89	0.00	4.00	2.28	4.58

**Table 2:** ANOVA for consequences variables in the study, taking into consideration the levels of global burnout.

	Global Burnout Levels							
	Very Low (*n* = 7) (≤P10) M (*SD*)	Low (*n* = 40) (≤P33) M (*SD*)	Medium (*n* = 117) (≤P66) M (*SD*)	High (*n* = 141) (<P90) M (*SD*)	Profile 1 (*n* = 45) (≥P90 and <P90 Guilt) M (*SD*)	Profile 2 (*n* = 22) (≥P90 and ≥P90 Guilt) M (*SD*)	F_(5,366)_	η_p_^2^
Depression	2.71 (3.77)	3.39 (3.10)	3.66 (3.48)	6.90 (6.03)	8.82 (6.92)	13.36 (9.83)	17.51 *	0.193
Psychosomatic disorders	0.49 (0.44)	0.61 (0.49)	0.64 (0.49)	0.86 (0.58)	1.12 (0.71)	1.52 (0.77)	13.11 *	0.152
Alcohol intake	0.86 (1.46)	0.70 (0.76)	0.92 (1.02)	1.23 (1.00)	1.29 (1.06)	1.68 (1.32)	4.25 *	0.054
Tobacco intake	0.00 (0.00)	0.10 (0.38)	0.30 (0.73)	0.39 (0.83)	0.84 (1.26)	1.02 (1.33)	6.23 *	0.078

* *p* < 0.001, Note 1. Very low level of global burnout are values equal to or lower than the 10th percentile—according to SBI Manual values, Gil-Monte [17]—on the mean of the 15 items from the subscales of enthusiasm towards the job (reversed; 5 items), psychological exhaustion (4 items), and indolence (6 items). Low levels are values higher than the 10th percentile and equal to or lower than the 33th percentile. Medium levels are values higher than the 33th percentile and equal to or lower than the 66th percentile. High levels are values higher than the 66th percentile and lower than the 90th percentile. Profile 1 are values equal to or higher than the 90th percentile on global burnout together values lower than the 90th percentile on the guilt subscale. Profile 2 are values equal to or higher than the 90th percentile on global burnout together values equal to or higher than the 90th percentile on the guilt subscale.
